# Evaluation of systemic inflammatory response syndrome-negative sepsis from a Chinese regional pediatric network

**DOI:** 10.1186/s12887-018-1364-8

**Published:** 2019-01-08

**Authors:** Yuanyuan Wang, Xiaofei Lin, Hongni Yue, Niranjan Kissoon, Bo Sun, Collaborative Study Group for Pediatric Sepsis in Huai’an, Bo Sun, Yuanyuan Wang, Hongni Yue, Xiaofei Lin, Bing Li, Chunlin Yu, Xiaochun Yang, Chunming Shan, Yujin Fan, Maotian Dong, Li Hao, Yixing Zhang, Wenlong Lin, Xiaofeng Zuo, Tingting Yin, Ping Su, Yongbo Heng, Jinzhong Xu

**Affiliations:** 1 Departments of Pediatrics and Pediatric Critical Care, Children’s Hospital of Fudan University, 399 Wanyuan Road, Shanghai, 201102 China; 2 Departments of Pediatrics and Pediatric Critical Care, Huai’an Women and Children’s Hospital, Huai’an, 223002 Jiangsu China; 3 Department of Pediatrics, University of British-Columbia and BC Children’s Hospital, Vancouver, BC Canada

**Keywords:** Infection, Outcome, Pediatrics, Sepsis, Systemic inflammatory response syndrome, Therapy

## Abstract

**Background:**

The identification of sepsis in children varies depending on the definition used. Our purpose was to compare clinical data and outcome of atypical sepsis, manifested as having sepsis but not fulfilling the criteria of systemic inflammatory response syndrome (SIRS-negative sepsis, SNS), in children based on the modified Angus criteria with those of sepsis (S) and severe sepsis (SS) based on the international consensus criteria.

**Methods:**

Pediatric departments in 11 regional city and county referral hospitals with emergency and intensive care settings in Huai’an serving for 843,000 children participated in a parallel multicenter prospective survey. Clinical data registry was used to recruit those who fulfilled the diagnostic criteria for pediatric sepsis from all admissions (*n* = 27,836) from 28 days to 15 years old, from September 1, 2010 to August 31, 2011.

**Results:**

A total of 1606 children met the criteria for pediatric sepsis and were divided into three groups: S, (*n* = 1377), SS (*n* = 153, including 32 septic shock), based on the consensus definition criteria, and SNS (*n* = 76) based on the modified Angus criteria. Most deaths (38/54, 70.3%) occurred within three days of admission. The SNS mainly occurred in infants and was associated with cardiopulmonary and neurologic dysfunction without satisfying the SIRS criteria.

**Conclusions:**

SNS differed from SS in that it predominantly affected infants and manifested with cardiopulmonary and neurologic dysfunction. There were no laboratory variables which were useful in identification of SNS, or predicting response to therapy or outcome.

## Background

Severe sepsis and septic shock in infancy and early childhood remain a significant cause of morbidity and mortality worldwide. The World Health Organization statistics suggests that at least 50% of death in children less than 4 years old in developing and underdeveloped countries and regions may be associated with sepsis [1–3]. Even in the developed countries, pediatric sepsis remains an important burden with regard to its incidence and outcome [4, 5]. The mortality rate of pediatric sepsis from pediatric intensive care units (PICUs) of developing countries is higher than 50% [6, 7], and is related to many factors including availability and affordability of care.

Studies in China showed that the frequency of sepsis in children in PICUs was 7.4–26.2%, and the associated mortality rate was 17–31% [8]. Moreover, among those children with acute hypoxemic respiratory failure and acute respiratory distress syndrome with sepsis as underlying pathologies had a very high mortality (> 70%), relative risk of death was 3–4 times higher than those without sepsis, and affecting mostly in those under 6 years old [9–11]. The identification of sepsis was based on the presence of systemic inflammatory response syndrome (SIRS) and presumed infection [12]. However, recently it is recognized that using the SIRS criteria one in eight cases of sepsis may be missed [13]. These cases while not satisfying SIRS criteria (SIRS negative) are identified by physicians by being infected or suspected of having an infection and organ dysfunction (the modified Angus criteria, [14]). Thus they are considered to have SIRS negative sepsis (SNS, [13]), synonymous as Angus-criteria sepsis, or atypical sepsis. While both criteria have gained acceptance, there was little data on the clinical course, laboratory data, therapy and outcome based on the criteria used for diagnosis of pediatric sepsis, severe sepsis (SS) and SNS, especially from a regional pediatric emergency service for all children’s population from post-neonatal infancy.

In our previous multicenter prospective survey of 1606 cases of sepsis and severe sepsis [15], we identified a group of patients with infection who did not satisfy the international criteria for sepsis [12] but was diagnosed as having sepsis based on the modified Angus criteria [14], hence we consider these patients to be SNS. The purpose of present study was to compare these patients using the modified Angus criteria with those of sepsis and SS diagnosed using the international criteria with regard to clinical and laboratory findings, treatment and outcomes. We hypothesized that SNS may have different manifestation from but similar clinical risks to SS, and should be treated with the same strategy and particular attention.

## Subjects and methods

### Study population and inclusion criteria

**Fig. 1:**
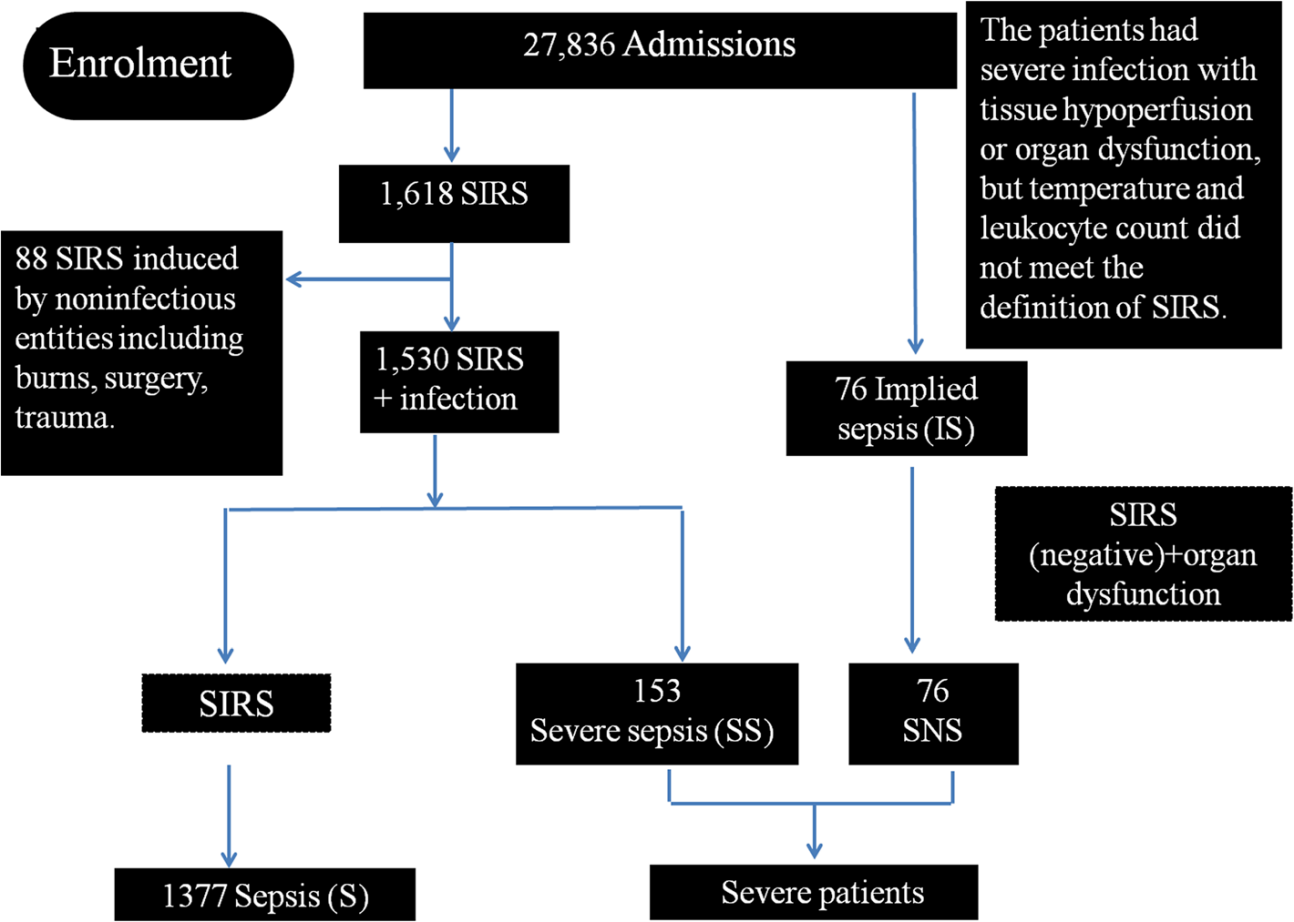
Distribution of patients and groups analyzed in the Huai’an Sepsis Study. Patients initially presented as systemic inflammatory response syndrome (SIRS)-negative sepsis (SNS), or modified Angus criteria for sepsis definition (severe infection with tissue hypoperfusion or organ dysfunction, but with fewer than two criteria of conventional criteria for SIRS), S (sepsis, equal to or more than two SIRS criteria), and severe sepsis (SS, sepsis with acute respiratory distress syndrome, cardiovascular organ dysfunction, or two or more other acute organ dysfunctions), according to the worst sepsis stage reached during hospitalization

This descriptive prospective, cross-sectional study was performed in Huai’an city, Jiangsu province in China, a semi-agronomic prefectural region, with 50% rural residents of a total population of 5.4 million, which was similar to the national average level of economic development as judged by the gross domestic production, resident income, and health care levels. To ensure quality of the survey, a collaborative study group was established among pediatric departments from 10 level II and III county and city general hospitals and a women and children’s hospital, of Huai’an region, consisting of a total of 541 ward beds and 16 PICU beds which were located in two tertiary care hospitals. The rural residents were subjected to the New Rural Cooperative Medical Scheme, a universal health insurance policy since 2010 which enabled prompted emergency and admission to care. Details of study protocol and compliance of investigator performance are reported elsewhere [15], and the patient enrolment and eligible for data analysis are shown in Fig. 1.

This study was coordinated by Huai’an Women and Children’s Hospital in collaboration with Children’s Hospital of Fudan University following approval of the study protocol by local ethics committees/institutional review boards [15]. To ensure quality of the study, training course for clinical staffs participating the study was provided prior to the commence of the survey, and case record form of each patient enrolled in the study was prospectively monitored by local co-investigator. At the conclusion of the treatment or in case of death, the case record form was completed, and completeness and accuracy checked by both co-investigator and staffs of the coordination center.

All children, 28 days to 15 years old, who were consecutively admitted to the participating hospitals from September 1, 2010 to August 31, 2011, were actively screened daily for the presence of sepsis (S), SS or septic shock using a diagnostic tool adopted from the international definitions for pediatric sepsis and organ dysfunction [12]. Patients were prospectively enrolled in the study when onset of S or SS was suspected based on the international guidelines and or was clinically suspected by attending physicians based on suspected or proven infection and organ dysfunction (modified Angus criteria for pediatrics) [14]. Severe sepsis was defined as sepsis with respiratory failure and/or acute respiratory distress syndrome, cardiovascular organ dysfunction, or two or more other acute organ dysfunction. Septic shock was defined as cardiovascular dysfunction or the presence of altered peripheral perfusion despite optimal fluid resuscitation [15–20]. The modified Angus criteria defined SNS as patients who does not meet the international consensus criteria but has a suspected or proven infection with at least one organ dysfunction or failure [14].

For each patient who satisfied the international guidelines for S and SS, clinical management and outcome were reported elsewhere [15]. For this study, the clinical management and outcome of children with SNS was reviewed for comparison. In addition, physical examination of peripheral perfusion, hematological and biochemical variables from peripheral blood samples were also recorded for site of infection, isolated pathogens, antibiotic therapy (prophylactic, based on resistance), and organ dysfunction/failure. These included total proteins, albumin, total bilirubin, aspartate aminotransferase, lactate dehydrogenase, serum creatinine, creatine kinase-myocardial band isoenzyme and blood routine examination during hospitalization. Values of these parameters in the first few days of study enrolment were compared among the patients in group with diagnosis of sepsis (S), SS [12, 15, 21] and SNS [14].

### Statistical analysis

Statistical analysis was performed using SPSS software v. 16.0. Continuous variables are summarized as median and interquartile range (IQR) or range, or as means and standard deviation (SD). Categorical data are presented as numbers and proportions in percentage. Comparisons of continuous variables are performed with the use of analysis of variance followed by Student-Newmann-Keuls post hoc test for between-group differences, or by Student t test, and comparisons of categorical variables are performed with Chi square test. A *P* value < 0.05 was considered statistically significant.

## Results

### Study population

Of the 27,836 children admitted to the 11 participating hospitals during the study period, a total of 1606 (5.8%) cases met the sepsis criteria [12]. In the end, a total of 153 SS and 76 SNS cases were included in the database. Table 1 illustrates baseline data and clinical characteristics of the three groups at study entry. Of the 1606 cases, age distribution was as follows: 57.5% in 1 month to < 2 years, 23.3% 2–4 years, 9.5% 5–7 years, and 9.5% 8–14 years old. The incidence of sepsis was the highest in children under five years old (*n* = 1297, 80.8%) with an overall higher incidence in boys than in girls in every group. In SNS group, most (65/76, 85%) of cases had sepsis onset below one year old. The proportion of patients admitted to the PICU was 110 (8.0%) of S, 116 (75.8%) SS and 45 (59.2%) SNS. The duration of hospital stay for all sepsis patients was 8.7 ± 3.0 [median (range) 8 (1–38)] days in S, 11.2 ± 10.6 [10 (1–70)] days in SS and 13.2 ± 6.7 [12 (2–36)] days in SNS. The hospital costs were 3408 ± 2986 [2611 (150–38,713)] Chinese Yuan (CNY) in S, 8884 ± 8230 [6620 (136–50,763)] Yuan in SS and 7916 ± 5193 [6315 (1079-27,964)] Yuan in SNS.

**Table 1:** Baseline data and clinical characteristics of the patients at study entry and outcome

Variables	Sepsis^a^	SS^a^	SNS	*p*
Case, *n*	1377	153^b^	76	
Age, year, mean (median)	3.3 (2.0)	1.6 (0.6)	0.7 (0.3)	0.000
Gender, boys (girls), *n* (n)	904 (473)	95 (58)	50 (26)	0.678
Rural residents, *n* (%)	843 (61.7)	122 (79.7)	62 (81.6)	0.000
Temperature, mean ± SD, °C	39.0 ± 0.7	38.3 ± 1.4	37.2 ± 0.6	0.000
Underlying diseases, *n* (%)	55 (4.0)	55 (35.9)	22 (28.9)	0.000
C-reactive protein (CRP)	36.9 ± 55.0	32.5 ± 58.5	6.7 ± 18.5	0.000
Sources of infection, *n* (%)				
Lower respiratory tract	418 (30.4)	98 (64.0)	68 (89.5)	0.000
Upper respiratory tract	414 (30.1)	0	0	0.000
Central nervous system	145 (10.5)	20 (13.1)	5 (6.6)	0.000
Unknown	265 (19.2)	13 (7.4)	1 (1.3)	0.000
Gastrointestinal system	67 (4.9)	17 (11.1)	1 (1.3)	0.000
Blood stream	49 (3.6)	4 (2.6)	1 (1.3)	0.000
Soft tissue	18 (1.3)	1 (0.7)	0	0.000
Mental status, *n* (%)				
Irritability	21 (1.5)	34 (22.2)	21 (27.6)	0.000
Convulsions	147 (10.7)	32 (20.9)	5 (6.6)	0.000
Twitches	6 (0.4)	3 (2.0)	0	0.000
Decreased level of consciousness	275 (20.0)	121 (79.1)	42 (55.3)	0.000
Organ or system dysfunction, *n* (%)				
Cardiovascular	0	32 (20.9)	42 (55.3)	0.000
Respiratory	0	58 (37.9)	2 (2.6)	0.000
Coagulation	0	8 (5.2)	1 (1.3)	0.000
Electrolyte imbalance	15 (1.1)	22 (14.4)	3 (3.9)	0.000
MODS	0	27 (17.6)	0	
Deaths, *n* (%)	1 (0.1)	53 (34.6)	1 (1.3)	0.000

Group definitions: Sepsis, sepsis; SS, severe sepsis; SNS, SIRS-negative sepsis

Definition of abbreviations: MODS, multiple organ dysfunction syndrome

^a^Values are taken from reference [15]; ^b^Numbers include 32 septic shock

### Clinical manifestations

An average time interval between the onset of signs and symptoms of infection to the initial diagnosis of SS and SNS was 6.1 ± 8.7 (median [IQR], 3 [2–7]) and 5.4 ± 5.1 (4 [3–6]) days, respectively [15]. In 578 (36%) patients who met SIRS criteria the initial abnormal temperature and/or white blood cell counts (WBC) at enrolment was transient and resolved by 48 h after enrolment [15]. Most of these patients received first treatment at local clinics, or received oral medication at home at the onset of symptom. In SNS group there were 36 with tachycardia, 68 high mean respiratory rate, none with core body temperature > 38.5 °C or < 36 °C, and none with peripheral WBC significantly out of the ranges for ages. Average levels of the peripheral WBC was 15–20 × 10^9^/L in S, with significant reduction from study entry to the period during 24–72 h of treatment whereas there was no significant changes (10–15 × 10^9^/L) for both SS and SNS groups.

Most patients in S group had mild depression of consciousness, however in SS and SNS patients, delirium, somnolence and coma were often found. Loss of consciousness and coma were associated with poor prognosis with a case fatality rate of 52.2% for coma (24/46) and 92.8% for deep coma (13/14) in the three groups. Most of the deaths occurred in SS except one each in S (aplastic anemia) and SNS. Infections of the lungs, central nervous system, and digestive system often led to SS or SNS. Nearly 90% SNS had respiratory tract infections (severe pneumonia), 55.3% (42 cases) severe cardiovascular system dysfunction, and 8% (6 cases) severe encephalitis (including purulent meningitis and viral meningitis).

A total of 51 cases (1 in SNS, 50 in SS) was diagnosed as respiratory failure, but in 7 cases escalated treatment such as ventilator use was declined by parents. The ventilator-use time was 3.3 ± 3.4 [median (range) 2.0 [1–12]] days. Fifty-five patients of all sepsis died, of which 5 patients died during the 30-day follow-up period after discharge including one patient died of aplastic anemia, thus the mortality of sepsis was 3.4% (54/1606). Besides, 54 cases all died of the condition of severe sepsis (in SS and SNS group), thus the case specific mortality rate of severe sepsis was 23.6% (54/229), and 38 cases with severe sepsis (70.3%, 38/54) died within three days from admission.

No evidence of specific variables for predicting organ dysfunction in SS and SNS from physical examination, or from the values of biochemical and cytological measurements, was found. The values of C-reactive protein in SNS were significantly lower than S and SS but skewed with very low average values (and median 14.4, range 0–390 for S, 4.2, 0.4–299 for SS and 1.6, 0.4–119 mg/100 mL for SNS), which also coincided with their clinical feature. The details in each group are compared in Table 2.

**Table 2:** Medications in the three groups

	Sepsis^c^	SS^c^	SNS	*p*
Cases, *n*	1377	153^d^	76	
Antimicrobial therapy				
pre-hospital, *n* (%)	1054 (76.5)	123 (80.9)	65 (86.7)	0.101
during, *n* (%)	1354 (98.3)	148 (96.7)	76 (100.0)	0.177
within 2 h, *n* (%)	1243 (91.7)	132 (88.6)	69 (90.8)	0.422
time, days mean ± SD	8.4 ± 4.6	10.3 ± 7.9^a^	12.4 ± 5.5^ab^	0.000
Other therapy				
GS, *n* (%)	500 (36.4)	120 (79.5)^a^	57 (75.0)^a^	0.000
VA, *n* (%)	23 (0.5)	103 (67.3)^a^	46 (60.5)^a^	0.000
IA, *n* (%)	3 (0.2)	92 (60.1)^a^	63 (82.9)^ab^	0.000
Sedatives, *n* (%)	100 (7.3)	109 (71.2)^a^	59 (77.6)^a^	0.000
IVIG, *n* (%)	52 (3.8)	49 (32.2)^a^	19 (25.0)^a^	0.000

Group definition: Sepsis, sepsis; SS, severe sepsis; SNS, SIRS-negative sepsis

Definition of abbreviations: pre-hospital, antimicrobial therapy was started prior to admission; during, antimicrobial therapy was given during hospitalization; within 2 h, intravenous antimicrobial therapy was started within 2 h of recognizing sepsis; time, the entire period of antimicrobial treatment; GS, glucocorticosteroids; VA, vasoactive agents; IA, inotropes; IVIG, intravenous immunoglobulin

Statistical analysis: ^a^ *p* < 0.01 vs, S; ^b^ *p* < 0.01 vs, SS

^c^Values are taken from reference [15]; ^d^Numbers include 32 septic shock

### Medications

In 77.6% of all patients, antibiotics were given in the local level I and II hospitals and health care clinics before admission, and 97.4% of which received in the first 2 h after admission, with 76.8% of the antibiotics treated as combined regimen. Cephalosporins and penicillins were the most frequently provided. Furthermore, significantly more patients in SS and SNS received antibiotics before admission (*P* < 0.001). The use of glucocorticosteroids was high, especially in SS (81.8%) and SNS (71.1%), and non-survivors had 79.3% treated with dexamethasone. The average duration of each course was 3.2 ± 3.0 (median (range) 2 (1–19)) days, in a range of dosage 0.1–0.3 mg/kg/day. In 11% of all patients received vasoactive drugs (*n* = 172), dopamine was the most frequently used (*n* = 142). Sixty-one percent (*n* = 106) of the survivors in SS and SNS groups received vasoactive drugs in contrast to 79.6% (*n* = 43) of the non-survivors. Intravenous immunoglobulin was provided in 120 cases (7.5% of all patients).

## Discussion

In this parallel, multicenter prospective survey in Huai’an we found that the clinical features and outcomes of children presenting with SS and SNS share both diversity and similarities that warrants a uniform approach to diagnosis and treatment. SNS was mainly found in infants with infection of respiratory system and clinically manifested as cardiopulmonary and neurologic dysfunction. In contradiction to SS none of the SNS infants satisfied SIRS criteria. These differences may be caused by viral infection and/or under-developed immune system in response to the infection. However, no laboratory variables were found to identify SNS, or to judge response to therapy or predict outcome.

The international consensus criteria were entailed to early recognition of sepsis [12]. Early recognition and treatment of sepsis can decrease mortality [19, 20]. However, the SIRS criteria have low specificity to identify infected patients at risk of exacerbation to severe sepsis or septic shock [22–24] and may miss one in eight adult patients with sepsis [13]. In our study, 76 (4.7%) cases of SNS had normal temperature and WBC, and did not meet SIRS criteria for presumed sepsis. This may be similar to the group identified by Kaukonen and colleagues as having sepsis but not meeting the international consensus criteria [13], and provides further evidence that using the SIRS criteria for sepsis screening is suboptimal [13–16]. In addition, in resource limited environments the use of SIRS for the criteria of sepsis may result in about 33% false-positives and has led to seeking alternatives to the traditional SIRS criteria in these settings [25]. We speculate that SIRS may be altered by response to medications such as antibiotics for a limited period, especially at level I clinics in this cohort (Table 2). However, if the SIRS persists, then clinical deterioration follows, leading to severe sepsis [26]. Patients in the SNS group had no history of SIRS, or any other explanation except sepsis for their clinical status, a phenomenon which has been previously reported [27]. It revealed that pediatric sepsis patient population may not share common clinical features and laboratory findings but SIRS-negative as a small proportion as those of adults [13, 14, 28].

The SS group was associated with a higher incidence of hypoxia and multiple organ dysfunction. In contrast in SNS, there were 90% as respiratory tract infections, 55.3% severe cardiovascular system dysfunction requiring vasopressors and inotropes, and 8% severe encephalitis, though proportion of organ system dysfunction and deaths were lower (Table 1). The clinical courses were similar between SS and SNS, but their mechanisms related to organ system dysfunction and deaths are yet to be determined. The main implication of our findings is that the approach to management should be similar in children presenting with SS or SNS in light of the absence of SIRS criteria and no laboratory data to differentiate SS from SNS. While SIRS may be practically used for early screening for systemic infection in children, for the absence of SIRS criteria in young children, especially in infants, it does not preclude risks of developing SS or SNS.

Our study has limitations. Because there was no observation room in the local level I hospitals, outpatients with the possibility of developing sepsis may be missed because of an insufficient observation period. However, due to universal coverage of health insurance mandate, a robust regional transport system which also warranted prompt access to the level II and III hospitals, and the active admission policy and practice in the 11 participating hospitals, through regional infrastructure of pediatric emergency care, for the patients suspected of severe infection and/or organ dysfunction, few cases would be missed.

## Conclusion

We found in a large sample of pediatric sepsis that a small group of patients who clinically manifested as implied, SIRS-negative, sepsis and were not identified using the international consensus criteria. These patients were infants predominantly with cardiopulmonary and neurologic dysfunction. Thus the findings of changes in mental status and cardiopulmonary morbidities in infants without SIRS criteria may indicate SNS and should prompt early intervention. Validation of our findings will rely on a larger cohort from other locations.

## Abbreviations

CRP: C-Reactive protein
PICU: Pediatric intensive care unit
SIRS: Systemic inflammatory response syndrome
SNS: SIRS-Negative sepsis
SS: Severe sepsis
WBC: White blood cell counts
